# Treadmill running suppresses the vulnerability of dopamine D_2_ receptor deficiency to obesity and metabolic complications: a pilot study

**DOI:** 10.20463/jenb.2018.0023

**Published:** 2018-09-30

**Authors:** Jinkyung Cho, Donghyun Kim, Jungmoon Jang, Jeonghyeon Kim, Hyunsik Kang

**Affiliations:** 1 College of Sport Science, Sungkyunkwan University, Suwon Republic of Korea

**Keywords:** Dopamine, obesity, treadmill running, metabolic disorders, mice, high-fat diet

## Abstract

**[Purpose]:**

To investigate the effect of treadmill running on D_2_R deficiency related susceptibility to high fat diet (HFD )-induced obesity and its metabolic complications.

**[Methods]:**

D_2_R-/-and +/-mice were obtained by backcrossing D_2_R+/-heterozygotes on wild type (WT) littermates (C57BL/6J background) for >10 generations. Mice were randomly assigned to 1) WT mice with standard chow (SC) (WT+SC); 2) WT mice with high-fat diet (WT+HFD); 3) WT mice with high-fat diet plus exercise (WT+HFD+EX), 4) heterozygous (HET) D_2_R mice with SC (HET+SC); 5) heterozygous D_2_R mice with HFD (HET+HFD); and 6) heterozygous D_2_R mice with HFD plus exercise (HET+HFD+EX). In addition, mice assigned to EX groups were subjected to running on a motor-driven rodent treadmill with a frequency of 5 days per week.

**[Results]:**

After a 10-week HFD treatment, HET D_2_R (+/-) mice exhibited significantly higher values for hepatic steatosis (*p*<0.001), areas under the curves (AUCs) for the glucose tolerance test (GTT) and the insulin tolerance test (ITT) (*p*<0.001 & *p*<0.001 respectively), serum leptin (*p*=0.005) and total cholesterol (TC ) (*p*=0.009), in conjunction with decreased locomotor activity (*p*=0.031), compared to HET mice exposed to standard chow. However, these HFD-induced elevations in hepatic steatosis (*p*<0.001), AUCs for GTT and ITT (*p*=0.032 & *p*=0.018, respectively), serum leptin (*p*=0.038) and TC (*p*=0.038) were significantly alleviated after 10 weeks of treadmill running.

**[Conclusion]:**

The current findings of the study provide experimental evidence of treadmill running as an effective and non-pharmacologic strategy to treat the susceptibility of brain D_2_R deficiency to HFD-induced obesity and metabolic disorders.

## INTRODUCTION

Dopamine (DA) in the central nervous system plays a key role in control of energy homeostasis via a modulation of eating behaviors and/or locomotor activity^1^. Lower sensitivity of the reward circuitry involving dopaminergic neural substrates has been blamed for obesity associated with compulsive eating behaviors. In support of the reward deficit theory, evidences from both clinical and genetic studies have been accumulated to demonstrate that like drug addicts, obese people tend to have altered expression of DA D_2_ receptors (D_2_R) in specific brain areas activated by food-and drug-related cues^2-4^. Likewise, it has been also suggest that reduced D_2_R expression may result in decreased locomotor activity^5^, shifting energy balance toward greater energy conservation and storage^6^. Furthermore, DA signaling is crucial for leptin-induced anorexic effect in fasted mice^7^, suggesting a functional link between D_2_R and leptin signaling in control of energy homeostasis^8^. Together, the susceptibility of decreased D_2_R to obesity and its metabolic complications remains to be further explored.

Complete knockouts (KO) often induce more severe abnormalities than knockdowns (KD), provoking the question of whether a knockdown, more consistent with reduced rather than ablated D_2_R signaling, yields different results. In heterozygous (+/-) and homozygous (-/-) D_2_R mice, D_2_R messenger RNA levels are reduced to 55% and 3%, respectively, of wild type (WT) littermates^5^. In addition, homozygous D_2_R (-/-) mice have several pathologic manifestations, including chronic hyperprolactinemia, pituitary hyperplasia, a moderately decrease in melanocyte-stimulating hormone (MSH) content^9^, growth retardation^10^, and impaired glucose homeostasis^11^. Those pathological characteristics are likely to act as potent compounders in elucidating the role of brain D_2_R deficiency in the etiology of high-fat-diet (HFD)-induced obesity. Therefore, we believe that the use of D2R heterozygous (+/-) rather than null mice is a more appropriate choice to reflect the susceptibility of D2R deficiency to obesity in a clinical setting.

On the other hand, it has been well established that physical activity per se provides a protection against obesity and metabolic complications in Otsuka Long-Evans Tokushima fatty (OLETF) rats^12^ and WT mice^13-14^. Yet, the therapeutic potential of exercise training against the susceptibility of brain D_2_R deficiency to obesity and its metabolic complications has received little attention. In this study, we used heterozygous D_2_R KD (+/-) mice to investigate the effects of treadmill running on the susceptibility of D_2_R deficiency to HFD-induced obesity and its metabolic complications.

## METHODS

### Mice

D_2_R^−/−^ mice (B6;129S2-Drd2tmllow) were originally obtained from the Induced Mutant Resource at the Jackson Laboratory (Bar Harbor, ME). D_2_R^−/−^ and +/-mice were bred by backcrossing D_2_R^+/–^ heterozygotes with wild type (WT) littermates (C57BL6/J background) for >10 generations. Mice were housed in pairs and had free access to food and tap water at a pathogen-free animal care facility located at our institute, under controlled light (12:12 h light-dark cycles starting at 0800 hours), humidity (50%) and temperature (20~23℃) conditions.

**Fig. 1.**
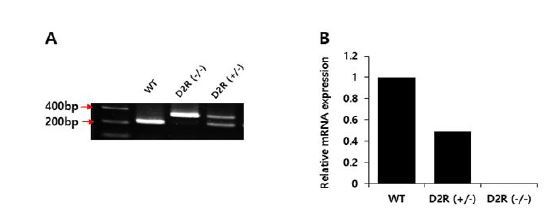
Dopamine D2 receptor genotyping (A) and mRNA levels (B). WT: wild type; D_2_R (-/-): dopamine D2 receptor (-/-) homozygotes; D_2_R (+/-): dopamine D2 receptor heterozygotes.

Genotyping was performed by polymerase chain reaction (PCR) using genomic DNA extracted from tail samples, QIAmp DNA Mini Kits (QIAGEN, Hilden, Germany), and DNA Taq polymerase (Thermo Scientific, Brookfield, Wisconsin, USA). Sequences of primer sets were: 5ʹ-TGA TGA CTG GGA ATG TTG GTG TGC-3ʹ (forward), 5ʹ-CTC CCC AGA GTT GTG GCA AAA GG-3ʹ (reverse), and 5ʹ-AGG ATT GGG AAG ACA ATA GCA G-3ʹ (reverse). The primers were designed to produce two DNA fragments of 329 bp and 221 bp for D_2_R^+/-^ and a single DNA fragment of 221 bp for WT (Fig. 1A). We also confirmed D_2_R mRNA levels for heterozygous and homozygous mice (Fig. 1B). The experimental protocol was approved by the Institutional Animal Care and Use Committee (IACUC) of Sungkyunkwan University School of Medicine (SUSM).

### Experimental design

After adapting to animal care facilities, mice were randomly assigned to (5 male and 5 female mice per group): 1) WT mice with standard chow (SC) (WT+SC); 2) WT mice with high-fat diet (WT+HFD); 3) WT mice with high-fat diet plus exercise (WT+HFD+EX), 4) heterozygous (HET) D_2_R mice with SC (HET+SC); 5) heterozygous D_2_R mice with HFD (HET+HFD); and 6) heterozygous D_2_R mice with HFD plus exercise (HET+HFD+EX). SC diet consisted of regular mice chow pellets containing 20% fat, 70% carbohydrates, and 10% protein in kcal (Purina Mills, Seoul, Korea) and lasted for 10 weeks. HF diet consisted of small pellets containing 60% fat (90% lard, 10% soybean oil), 20% carbohydrates, and 20% protein in kcal (D12492 Research Diet, New Brunswick, NJ) and lasted for 10 weeks.

Mice assigned to those EX groups were additionally subjected to running on a motor-driven rodent treadmill (Columbus Instruments, Columbus, OH, USA ) at a frequency of 5 days per week for 10 weeks. Each session of treadmill running began with a warm-up at a speed of 8 m/min for 5-min, followed by 40-min of running at 12 m/min, and ended with a 5-min cool down at 8 m/min.

All animals were allowed to eat ad libitum. Food intakes and body weights were recorded twice a week for the entire treatment period. Diets were well tolerated by the animals. Diet intervention applied in this study is widely used to induce obese mice.

### Real-time PCR

Total RNA samples from the striatum were extracted using total RNA extraction kits (Applied Biosystems, Foster City, California, USA) according to the manufacturer’s protocol. Quantification and purity assessment of extracted RNAs were conducted using a Thermo Scientific *NanoDrop 2000* spectrophotometer (Brookfield, Wisconsin, USA). Quantitative PCRs were performed with Taqman RNA-to-CT 1-step kits in an ABI Prism 7500 real-time System (Applied Biosystems). FAM-labeled TaqMan probes for mice were obtained from a commercial source (TaqMan Gene Expression Assays; Applied Biosystems). Gene expression was normalized to β-actin. Experiments were performed in triplicate for each sample per group. The 2^−ΔΔCt^ method was used for statistical analysis of real-time PCR results.

### Open field test

Locomotor activity (total lines crossed and line crosses in outer squares, in cm) was monitored on an open field apparatus made of white plywood and measuring 50 x 50 cm with a height of 35 cm. Mice were carried to the test room in their cages and handled at all times by the base of their tails. Mice were placed in the center of the open field and allowed to explore the apparatus for 5 min. Following the 5 min test, mice were returned to their cages whereupon the open field was cleaned with an ethanol-water solution and permitted to dry between tests. To assess the process of habituation to the arena, mice were exposed to the apparatus for 5 min on 2 consecutive days. Exploratory behaviors were recorded in terms of total distance (m) measured with a computerized video-tracking system (ANY-maze TM, Stoelting, USA).

Following the open field test, mice were anesthetized with a mixture of Alfaxan 80 mg/kg and Rompun 10 mg/kg, transcardially perfused with 1 × PBS. Mice were then sacrificed, and livers were quickly removed and flash frozen in liquid nitrogen for histological examination.

### Glucose and Insulin Tolerance Tests (GTT and ITT)

GTT was conducted with a bolus intra-peritoneal (IP) injection (1.5 g/kg body weight) of glucose (Sigma-Aldrich, St. Louis, MO) following a 16-hr fasting. Blood samples were collected from a cut at the tip of the tail before and 15, 30, 45, 60 and 120 minutes after glucose IP injection. ITT was conducted with an IP injection (1 U/kg body weight) of insulin (Sigma) following a 4-hr fasting. Blood sample were collected from a cut at the tip of the tail before and 15, 30, and 45 minutes after insulin injection. Serum blood glucose was measured using the One Touch II glucose meter (Life Scan, Milpitas, CA). Areas under the curve (AUCs) for GTT and ITT were calculated using the linear trapezoid method. GTT and ITT were conducted 1 week prior to scarification of mice.

### Biochemical assays

Plasma insulin concentrations were measured using a commercially available enzymatically-linked immunoassay (ELISA) kit (ALPCO, Salem, NH). Plasma leptin levels were measured using mouse ELISA kits (Millipore, Billerica, MA). Serum total cholesterol (TC) and triglyceride (TG) contents were measured using commercially available enzymatic kits (WAKO Chemicals USA, Inc., Richmond, VA).

### Histology

Liver tissues were embedded in paraffin and fixed in 10% formalin for 24 h for hematoxylin-eosin (H&E) staining. Liver samples were then sectioned using a microtome (CM3050S, Leica Microsystems, Nussloch, Germany) into 5 μm thick slices and stained with H&E for routine histological examination. A steatosis grade of 0-3 was assigned according to the Kleiner system^15^ which describes the extent of steatosis based on lipid accumulation in hepatocytes (0, <5%; 1, 5-33%; 2, 33-66%; and 3, >66%).

### Statistics

Results were expressed as mean ± SD. Two-way ANOVA followed by the least significant difference (LSD) post hoc test, was used where necessary to test significant interactions between genotypes (WT vs. HET), treatments (SC vs. HFD vs. HFD+EX) or their interaction. Statistical significance was set at *p* < 0.05. All statistical analyses were performed using the IBM SPSS-PC statistical software (version 24.0).

## RESULTS

### Treadmill running alleviated HFD-induced obesity and decreased locomotor activity

**Fig. 2.**
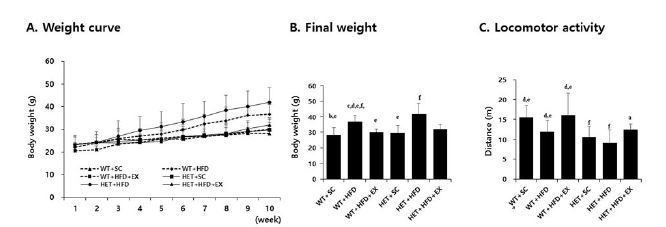
Weight curve (A), final weight (B), and locomotor activity (C). WT: wild type; SC: standard chow; HFD: high-fat-diet; EX: exercise training. a: significantly different from WT+SC, b: significantly different from WT+HFD, c: significantly different from WT+HFD+EX, d: significantly different from HET+SC, e: significantly different from HET+HFD, f: significantly different from HET+HFD+EX (*p*<0.05)

After a 10-week treatment, there were significant genotype (F(1,5)=6.178, P=0.020) and treatment (F(2,5)=13.882, P<0.001) effects on final weight, with no significant genotype by treatment interaction (F(2,5)=0.562, P=0.577). WT+HFD mice had a higher mean weight gain (75.8% vs. 22.7%, P=0.017) than WT+SC mice. Likewise, HET+HFD mice had a higher weight gain (86.8% vs. 28.1%, P=0.012) than HET+SC mice. On the other hand, the analysis of weight curves showed that HET+HFD mice gained consistently more weight over the course of a 10-week HFD than WT+HFD mice (Figures 2A-B). Liver histology showed a significant genotype by treatment interaction (F(2, 5)=19.270, P<0.001) on hepatic steatosis; HET+HFD mice had more severe fatty liver by 50.8% (P<0.001) than WT+HFD mice (Figures 4A-B).

With respect to locomotor activity, there were significant genotype (F(1,5)=19.538, P<0.001) and treatment effects (F(2,5)=9.815, P=0.001), with no significant genotype by treatment interaction (F(2, 5)=0.428, P=0.658). HET mice (SC+HFD) had significantly lower locomotor activity (P=0.008) than WT mice (SC+HFD). Likewise, WT+HFD mice had significantly lower locomotor activity (P=0.040) than WT+SC, with no significant difference in locomotor activity (P=0.261) between and HET+HFD and HET+SC mice (Figure 2C).

After 10 weeks of treadmill running, however, WT+HFD+EX and HET+HFD+EX mice had lower weight gains (P=0.035 & P=0.032, respectively) and less severe hepatic steatosis (P<0.001 & P<0.001, respectively) than WT+HFD and HET+HFD mice, respectively (Figures 2A-B), with no statistically significant difference (P=0.100 for body weight and P=0.507 for hepatic steatosis) between WT+HFD+EX and HET+HFD+EX mice. In addition, WT+HFD+EX and HET+HFD+EX mice had significantly higher locomotor activities (P=0.012 & P=0.031, respectively) than WT+HFD and HET+HFD mice, respectively. Together, the current findings of the study suggest a therapeutic potential of treadmill running in treating obesity phenotypes and decreased voluntary locomotion associated with D_2_R deficiency or HFD or a combination of D_2_R deficiency and HFD.

### Treadmill running alleviated HFD-induced metabolic complications

**Fig. 3.**
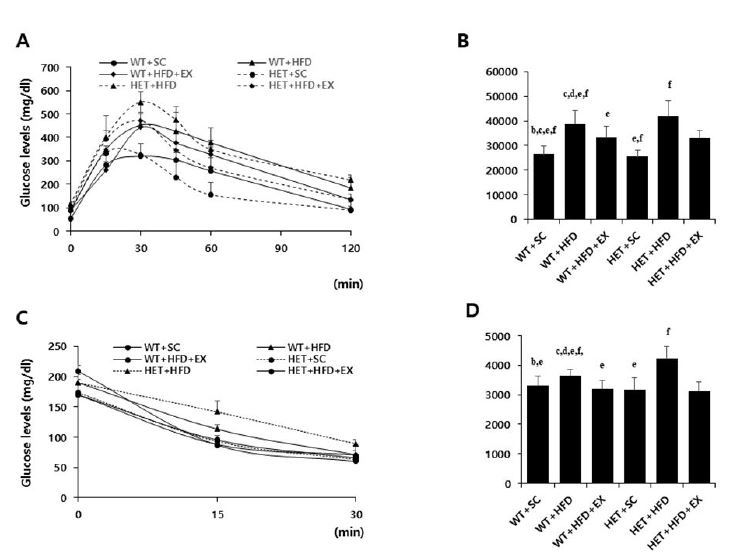
Glucose and insulin tolerance tests. WT: wild type; SC: standard chow; HFD: high-fat-diet; EX: exercise training. A: glucose tolerance test (GTT); B: area under the curve (AUC) of GTT; C: insulin tolerance test (ITT); D: area under the curve (AUC) of ITT. a: significantly different from WT+SC, b: significantly different from WT+HFD, c: significantly different from WT+HFD+EX, d: significantly different from HET+SC, e: significantly different from HET+HFD, f: significantly different from HET+HFD+EX (*p*<0.05)

After a 10-week treatment, there were significant genotype by treatment interactions on AUC for GTT (F(2, 5)=3.458, P=0.047) and AUC for ITT (F(2, 5)=10.078, P=0.001); HET+HFD mice had significantly higher AUC for GTT (P=0.007) and AUC for ITT (P<0.001) than WT+HFD mice (Figures 3A-D). Similarly, there was a significant genotype by treatment interaction on serum leptin (F(2, 5)=4.420, p=0.027); HET+HFD mice had significantly higher serum leptin levels (P=0.003) than WT+HFD mice. In addition, HET+HFD mice tended to have higher TC levels (F(2, 5)=3.303, P=0.057) than WT+HFD mice. However, there was no significant genotype (F(1, 5)=0.712, P=0.413) or treatment (F(2, 5)=0.317, P=0.734) or genotype by treatment interaction (F(2, 5)=2.683, P=0.103) on serum TG.

**Fig. 4.**
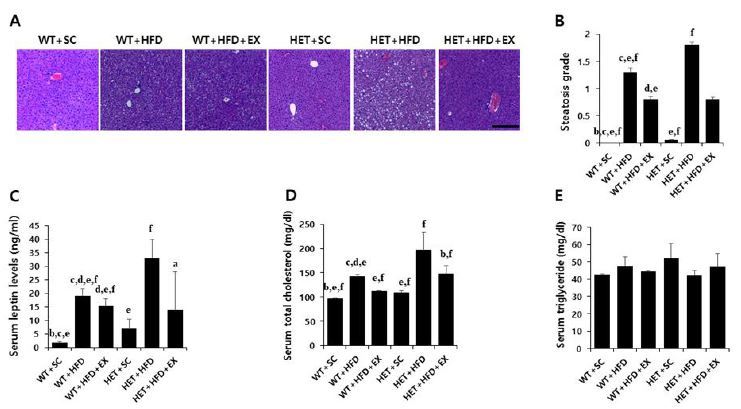
Liver histology, serum leptin and lipids. WT: wild type; SC: standard chow; HFD: high-fat-diet; EX: exercise training. A: liver histology; B: steatosis grade; C: serum leptin; D: total cholesterol (TC); E: triglycerides (TG). a: significantly different from WT+SC, b: significantly different from WT+HFD, c: significantly different from WT+HFD+EX, d: significantly different from HET+SC, e: significantly different from HET+HFD, f: significantly different from HET+HFD+EX (*p*<0.05)

After 10 weeks of treadmill running, however, WT+HFD+EX and HET+HFD+EX mice had significantly lower AUC for GTT (P=0.021 & P=0.032, respectively), lower AUC for ITT (P=0.011 & P<0.018, respectively), lower serum leptin (P=0.044 & P=0.038, respectively), and lower serum TC (P=0.035 & P=0.038, respectively) than WT+HFD and HET+HFD mice (Figures 3A-D & Figures 4C-D). Together, the current findings of the study suggest that treadmill running alleviates HFD-induced metabolic complications independent of D_2_R deficiency.

## DISCUSSION

In this study, we investigated the effect of treadmill running on HFD-induced obesity and metabolic complications in D_2_R HET mice and found that forced exercise training suppressed the susceptibility of D_2_R deficiency to obesity phenotypes and metabolic complications, including impaired glucose tolerance, hepatic steatosis, hyperleptinemia, and hypercholesterolemia.

### Treadmill running and HFD-induced obesity and decreased locomotor activity

With respect to the susceptibility of D_2_R deficiency to obesity phenotypes, the current findings of the study are in agreement with previous studies reporting altered DA neurotransmission in relation to diet-induced obesity in animals. By studying obesity-prone and obesity-resistant rats, Geiger et al.^16^ showed that decreases in basal and electrically-evoked DA release from the nucleus accumbens and dorsal striatum were linked to hyperplasia and increased body mass. In addition, basal and feeding-evoked dopamine release is exaggerated in several nuclei of the hypothalamus of obese Zucker rats^17-19^, whereas D_2_R expression is reduced in hypothalamic nuclei of obese animal models^20,21^ in the striatum of obese humans^22^. Together, the current findings suggest that impaired dopamine reward system in the control of feeding behaviors may play a role in the development of HFD-induced obesity phenotypes.

On the other hand, Kravitz et al.^23^ showed that mice with low D_2_R were less active than control mice, suggesting that decreased energy expenditure secondary to impaired dopaminergic system may contribute to the development of obesity. In this study, we do not have a clear explanation for the susceptibility of brain D_2_R deficiency to obesity phenotypes such as increased weight gain and higher hepatic steatosis, especially with respect to energy balance. In recent studies, however, mice with D_2_R deficiency had decreased voluntary locomotion without vulnerability to diet-induced obesity, indicating that the susceptibility of D_2_R deficiency to the obesity phenotypes is a consequence of decreased energy expenditure^5^. In this aspect, hyperleptinemia observed in the D_2_R HET mice of the current study is of particular interesting. As an adipocyte-derived hormone, leptin regulates not only feeding behavior but also locomotor activity via direct actions on mesolimbic dopaminergic system. Thus, it is possible that hyperleptinemia may reflect a compensatory response to decreased locomotor activity due to impaired mesolimbic dopaminergic system.

On the other hand, Kim et al.^8^ showed that D_2_R knockout (-/-) mice had a lean phenotype in conjunction with increased basal metabolic rate. In that study, they found that D_2_R knockout mice had elevated hypothalamic MCH mRNA expression along with enhanced hypothalamic leptin signaling, as compared to WT littermates, implying the potential role of D_2_R-mediated enhancement of hypothalamic leptin signaling for the lean phenotype and increased metabolic rate secondary to D_2_R deficiency. Contradictory to those findings reported by Kim et al.^8^, we found that D_2_R HET mice had significantly higher serum leptin levels, implying the possibility of impaired leptin signaling or leptin resistance associated with brain D_2_R deficiency. D_2_R is expressed in the hypothalamus, which is the homeostatic regulatory center of feeding behaviors via leptin signaling. In addition to leptin, insulin and ghrelin exert their impact on the midbrain dopaminergic system via an interaction between the homeostatic and reward circuits of feeding behaviors. Consequently, a further study would be necessary to produce a clear answer for the contradictory.

Beeler et al.^5^ showed that D2R KD mice had decreased voluntary locomotion with no difference in baseline food intake than WT mice. In that study, they found that the beneficial effects of voluntary exercise on glucose and insulin tolerance tests were found in WT mice but not in D_2_R KD mice. In addition, D_2_R KD mice tended to decrease food intake consumption after voluntary wheeling running. Together, those findings, including the current one, suggest that decreased brain D_2_R availability may shift activity-related energy expenditure, paradoxically in favor of greater energy conservation and storage and thereby promoting obesity associated with HFD. This may also help explain the motivational barrier often encountered by exercise programs targeting at overweight and/obesity.

On the other hand, treadmill running alleviated the susceptibility of D_2_R deficiency to obesity phenotypes. Both voluntary wheel-running^25,26^ and forced exercise^27,28^ are effective in improving health and reducing obesity in rodents fed a high-fat diet. In particular, the benefits of forced exercise have been well established for the obesity phenotypes and metabolic complications in the fatty OLETF rats^29,30^ as well as in the HFD-induced obese WT mice^31,32^. Forced exercise is also shown to increase tissue dopamine levels as well as dopamine synthesis and metabolism^33,34^. In the current study, we also found that treadmill running alleviated the obesity and metabolic complications in response to HFD observed in D_2_R HET mice.

### Treadmill running and obesity-induced metabolic complications

With respect to metabolic complications, we found that chronic exposure to HFD led to impaired glucose tolerance and hypercholesterolemia in both WT and D2R HET mice. In particular, we found that D_2_R HET mice had elevated serum leptin at baseline and hyperleptinemia after exposed to chronic HFD, as compared to WT mice, implying that impaired dopaminergic system is involved in leptin resistance.

Leptin is an adipocyte-derived hormone that circulates in proportion to body fat mass^35^. Although an anorexic effect of leptin is generally attributed to signal transduction at the level of the basomedial hypothalamus^36^, studies also suggest that leptin regulates feeding behavior and locomotor activity via direct actions on mesolimbic dopaminergic system^37,38^. In this aspect, we also found decreased locomotor activity in D_2_R HET mice. Therefore, it seems reasonable to speculate that impaired brain D_2_R availability and its consequence of leptin resistance may lead to decreased locomotor activity and/or increased feeding behavior in response to HFD, contributing to obesity and its related metabolic complications, including impaired glucose tolerance and hypercholesterolemia. However, the mechanism(s) by which impaired dopaminergic system leads to leptin resistance remains to be investigated in the D_2_R HET mice.

On the other hand, we found that treadmill running alleviated HFD-induced metabolic complications in D_2_R HET mice. With respect to the improved metabolic complications, it is well known that exercise training improves insulin and leptin resistance in conjunction with dyslipidemia by promoting glucose and fatty acid metabolism as well as enhanced mitochondrial biogenesis. In addition, it seems possible that the elevated baseline serum leptin observed in D_2_R HET mice may reflect a compensatory response to genetic knockdown of D_2_R, exacerbating to hyperleptinemia after exposed to chronic HFD. In this aspect, it is reasonable to speculate that exercise/training increases brain D_2_R expression, thereby alleviating hyperleptinemia as well as impaired glucose tolerance and hypercholesterolemia. However, it is unlikely that treadmill running normalizes D_2_R deficiency in this genetically D_2_R knockdown mouse model. Considering the fact that dopamine affects energy hemostasis via D_2_R-mediated hypothalamic leptin signaling^6,33^, therefore, whether or not treadmill running-induced metabolic complications are independent of D_2_R-mediated hypothalamic leptin/insulin signaling should be further investigated.

Several explanations can be given for the suppressive effect of treadmill running against increased susceptibility of brain D_2_R deficiency to obesity phenotypes and metabolic complications. First, exercise and/or training in the form of forced one rather than voluntary one combats obesity associated with D_2_R deficiency by shifting energy balance toward an increase in activity-induced energy expenditure. Alternatively, physical activity has been shown to increase tissue dopamine levels as well as dopamine synthesis and metabolism^27,28^. Increased dopamine neurotransmission increases locomotor activity, prolongs the duration of physical activity, and attenuates the development of fatigue from physical activity^34,35^. Animals genetically modified to over-express dopamine, such as dopamine transporter knockout mice, also show increased locomotor activity^36^. Likewise, rats selectively bred for increased aerobic capacity show increased striatal DA activity, decreased body mass, and increased running distances compared to rats selectively for low aerobic capacity^37^.

Second, HFD-induced obesity promotes multiple cellular processes that attenuate leptin signaling, leading to the development of leptin resistance. On the other hand, it is well known that exercise and/or exercise training can reverse leptin resistance associated with HFD-induced obesity in rodents^38,39^. Therefore, we suspect that exercise training-induced increases of leptin sensitivity may enhance leptin signaling on the ventral tegmental area^40^ and/or corticotropin-releasing factor pathway^41^, positively modulating behaviors that influence appetite, locomotor control, and body weight.

Third, exercise training-induced activation of dopaminergic systems may contribute to energy homeostasis^42^. Chen et al.^43^ showed that treadmill running prevented HFD-induced insulin resistance in conjunction with increases in tyrosine hydroxylase (TH), D_2_R expression, and dopamine levels in the ventral tegmental area-nucleus accumbens system of C57BL/6J mice. By comparing two contrasting rat groups selectively bred for high (HCR) or low (LCR) capacity running, Foley et al.^40^ showed that rats with HCR had higher levels of DR-D_2_ autoreceptor mRNA in the midbrain and higher levels of DR-D_2_ postsynaptic mRNA in the striatum than rats with LCR, implying that exercise/training can upregulate dopamine receptor expression. However, whether or not treadmill running normalizes D_2_R deficiency remains to be investigated, although it is unlikely in genetic knockdown D_2_R mice.

Taken together, those findings suggest that forced physical exercise is one of effective and non-pharmacologic ways to prevent/treat the susceptibility of brain D_2_R deficiency to decreased voluntary locomotion and HFD-induced obesity and metabolic complications^44,45^. Yet, the mechanism(s) by which the risk of D_2_R deficiency to obesity phenotypes is reversed by exercise training, especially in the prospective of an interaction between leptin and dopamine signaling pathways in the hypothalamus, should be investigated in a future study. In addition, we failed to obtain the data regarding chow consumption and/or locomotion, a key determinant of daily energy balance. Consequently, we do not know whether or not the D_2_R deficiency to obesity is the consequence of either increased energy intake or decreased energy expenditure or both.

In conclusion, we found that after exposed to a 10-week HFD, D_2_R deficiency mice tended to gain more body weight in conjunction with more severe hepatic steatosis and glucose intolerance and hyperleptinemia, as compared to WT mice. On the other hand, we also found that those obesity and its related metabolic complications observed in the D_2_R deficiency mice were significantly alleviated after 12 weeks of treadmill running. Therefore, the current findings of the study suggest that forced exercise would be a strategic option against the susceptibility of brain D_2_R deficiency-induced motivational barrier to physical inactivity and subsequent consequences of obesity and metabolic complications.
